# Reducing Postbreast Reconstruction Infection Rates: Our Journey to Achieving Low Postoperative Infection Outcomes

**DOI:** 10.7759/cureus.76556

**Published:** 2024-12-29

**Authors:** Beshoy Meglaa, Badreldin Mohamed, Mina Tawfeek, Katherine Cerra, Hassan Abdulrahman, Ahmed Abdel-ghani, Abdalla Hassan, Mohamed Shaaban

**Affiliations:** 1 General Surgery, James Cook University Hospital, Middlesbrough, GBR; 2 Trauma and Orthopaedics, North Cumbria Integrated Care NHS Foundation Trust, Carlisle, GBR; 3 Breast Surgery, James Cook University Hospital, Middlesbrough, GBR

**Keywords:** antibiotic prophylaxis, drain management, implant breast reconstruction, infection control protocol, laminar flow in theatres, preoperative and postoperative measures, protocol of breast reconstruction surgery (pbrs), pseudomonas contamination, risk factors for ssi, surgical site infection(ssi)

## Abstract

Introduction: Breast surgeries are classified as clean procedures associated with a lower risk of post-operative infections; however, the reported infection rates post-breast surgeries are still significantly high. Surgical site infections (SSIs) are indeed one of the most common and serious complications following breast surgery.

Methodology: A retrospective study assessed the rate of SSIs post-breast reconstructive surgery after the implementation of the infection control protocol at James Cook University Hospital and Friarage Hospital from December 2022 to June 2024. Initial post-operative breast reconstruction cases at James Cook University Hospital and Friarage Hospital showed a high SSI rate of 23%, with Theatre 22 in James Cook University Hospital reporting 25%, despite adherence to infection control protocols.

Subsequent investigations identified Pseudomonas contamination in scrub water taps and inadequate airflow in Theatre 22 as potential sources. Corrective actions were implemented, including relocating surgeries to facilities with superior air filtration and forming a Protocol Reinforcement Team to ensure strict adherence to infection control measures and the Protocol of Breast Reconstruction Surgery (PBRS).

Data was collected from the information department of James Cook University Hospital retrospectively, looking at all patients who underwent breast reconstruction procedures from December 2022 to June 2024.

Patients who developed SSIs at the breast reconstruction implant site were identified by reviewing the patients' notes. The confirmed diagnosis as well as the classification and potential risk factors were identified retrospectively from the patients' notes as well. All patients included in the study were strictly adherent to the PBRS.

IBM SPSS Statistics for Windows, Version 21 (Released 2012; IBM Corp., Armonk, New York, United States) was used for both descriptive and inferential analyses of the data, and no adjustments were made for multiple comparisons.

Results: Among 31 patients (48 procedures), complications included delayed infection (n=1 procedure), drain exit site infection (n=1 procedure), skin necrosis (n=2 procedures), wound dehiscence (n=1 procedure), and seroma (n=1 procedure). We reported low infection rates to post-breast reconstruction with strict adherence to the PBRS (02.08%) in comparison to the current guidelines and published literature (up to 25%).

Conclusion: Despite improvements, SSIs remain a significant concern in implant-based breast reconstruction procedures. Adopting and adhering to a standardized PBRS are strongly recommended to further reduce complications and enhance patient outcomes.

## Introduction

Breast surgeries are generally considered clean procedures, which are typically associated with a lower risk of post-operative infections; however, the risk of surgical site infections (SSIs) can vary depending on factors like the type of surgery and the patient's health status [1]. The infection rate following breast reconstruction can be up to 25%, with about 70% of the infections occurring within the first 90 days after surgery. Infections of breast implants can lead to adverse outcomes such as implant removal, delays in administering adjuvant radiation and chemotherapy, and increased healthcare costs [2]. SSIs remain one of the most frequent and serious complications after surgery [2].

SSIs are common following breast surgeries, especially in procedures related to breast cancer. Chemotherapy and/or radiotherapy often compromise the immune response, making the tissue more liable to infections [3-5]. In addition, the other risk factors for SSIs after breast cancer surgery include high body mass index (BMI), smoking, alcohol use, diabetes, a history of other cancers, previous breast biopsies, immediate breast reconstruction, lack of prophylactic antibiotics on the day of surgery, and post-operative complications such as seroma and prolonged drainage duration [6-8]. Thus, there are many complicated factors involved in managing the risk of SSIs post-mastectomy.

National Health Service (NHS) National Mastectomy and Breast Reconstruction Audit 2011 showed that 20% of mastectomy patients and 25% of reconstruction patients required antibiotic treatment for a wound infection. One in two mastectomy patients (with or without immediate reconstruction) and a third of all delayed reconstruction patients required aspiration or drainage of a collection of fluid at their operative site [9].

Infection rates following mastectomy vary based on the type of reconstruction: implant-based reconstruction has 0.4% to 17% infection rate, while autologous flap reconstruction after mastectomy shows an SSI rate of 1% to 12% [7]. Staphylococci are the primary organisms responsible for SSIs following breast surgery, responsible for 60% of cases, with Gram-negative bacilli and anaerobes isolated in 40% of cases. In SSIs after implant-based breast reconstruction, gram-positive bacteria are isolated in 83% of patients, and of these, 49% involve methicillin-sensitive Staphylococci [5].

## Materials and methods

This retrospective study was conducted at James Cook University Hospital and Friarage Hospital from December 2022 to June 2024. The primary outcome of the study was to assess the rate of SSIs at breast reconstruction implants following breast reconstruction surgery at James Cook University Hospital and Friarage Hospital. The secondary outcomes are to identify the potential risk factors associated with SSI development in the study population, to investigate the potential infection control measures that can be implemented, and to compare SSI rates before and after the implementation of the Protocol of Breast Reconstruction Surgery (PBRS).

Initial post-operative breast reconstruction cases at James Cook University Hospital and Friarage Hospital showed a high SSI rate of 23%, with Theatre 22 in James Cook University Hospital reporting 25%, despite adherence to infection control protocols. These infection rates aligned with the 25% reported in the 2011 National Mastectomy and Breast Reconstruction Audit [9].

Subsequent investigations identified Pseudomonas contamination in scrub water taps and inadequate airflow in Theatre 22 as potential sources. Corrective actions were implemented, including relocating surgeries to facilities with superior air filtration and forming a Protocol Reinforcement Team to ensure strict adherence to infection control measures and the PBRS. 

This study exclusively included patients under the care of a breast consultant surgeon who strictly adhered to the PBRS.

The PBRS begins in the pre-operative assessment, ensuring negative methicillin-resistant Staphylococcus aureus (MRSA) swabs, and treatment of positive patients prior to attempting surgical reconstruction if necessary (this implementation conflicts with the hospital protocol). It also includes conducting medical photography preoperatively and post-operatively.

On the day of the operation, this protocol ensures patients take a morning shower with chlorhexidine, that they are well warmed, and that their temperature is normal.

Upon arrival at the theatre, intravenous (IV) antibiotic prophylaxis is administered at the time of anaesthetic induction (Teicoplanin 1.2 grams (g)), followed by 400 milligrams (mg) every 12 hours IV for two days.

A stop sign policy was enforced to minimize theatre traffic and activate laminar flow when available. Sterile Mayo’s tray for gowning and gloves were used and all team members wore masks covering the nose and mouth for five minutes before opening the surgical trays until the wound was closed. Surgeons and scrub nurses used hooded head covers, scrubbed using a scrub brush, kept the drape trolley away from the theatre tap, separated the drying towels, wore double gloves (e.g., reveal system), and used the closed-glove technique.

For the patient, we used nipple shields/glue, avoided peri-areolar incisions, and performed careful atraumatic dissection to minimize de-vascularized tissues and ensure careful haemostasis. We also avoided dissection into the breast parenchyma and skin-implant contamination.

The surgical team minimized the time of implant opening, repositioning, and replacement. They used an implant bag introducer even with expanders and employed a layered closure technique.

Theatre staff implemented a two-stage instrument tray system, which involved opening the second tray with re-prepping of the field and re-draping before handling the implant.

Surgical gloves were changed prior to handling the implant, after re-prepping and re-draping the field, using new instruments not used in the first stage.

All staff followed the department’s new policy and National Institute for Health and Care Excellence (NICE) guidelines for skin prep with chlorhexidine (2%).

An antibiotic solution was prepared using 1.2 g Teicoplanin, 240 mg Gentamicin, and 1000 millilitres (mL) saline for pocket irrigation and Implant washout/Mesh. The mesh was kept in the fluid for 30 minutes.

Post-operatively, we applied Pico dressings for seven days, with follow-up of the wound and drain removal arranged with the breast care nursing team. Drains were removed when output was less than 30 mL in 24 hours. The drain site was closed with nylon 4/0 using a local anaesthetic agent under aseptic technique.

Data was collected from the information department of James Cook University Hospital retrospectively, looking at all patients who underwent breast reconstruction procedures from December 2022 to June 2024.

SSIs developed by patients at the breast reconstruction implant site can be classified into superficial incisional SSIs, deep incisional SSIs, and organ/space SSIs. Superficial incisional SSIs occur in the first 30 days post-operatively, deep incisional SSIs occur within 30 days and can be up to one year post-procedure on fascial planes, and organ/space SSIs occur within 30 days and can be up to one-year post-procedure; infections typically beyond fascial planes were identified from reviewing the patient's notes. The confirmed diagnosis as well as the classification and potential risk factors were identified retrospectively from the patient's notes [10].

All patients included in the study were strictly adherent to the PBRS and this has been documented on the patient operation notes and post-procedure follow-up. No blinded review process was used.

IBM SPSS Statistics for Windows, Version 21 (Released 2012; IBM Corp., Armonk, New York, United States) was used for both descriptive and inferential analyses of the data, and no adjustments were made for multiple comparisons.

Patient consent was not needed in this study as it is an observational study and no patient’s confidential information was disclosed in the study; therefore patient confidentiality was maintained. Ethical approval is not needed as this is a retrospective study.

## Results

A retrospective study was conducted at James Cook University Hospital and Friarage Hospital from the 1st of December 2022 to the 30th of June 2024. Thirty-one patients (48 procedures) were included in this study, and all of them were female. Patients' demographics are shown in Table 1.

**Table 1:** Patients' demographics. n: Number of patients; %: percentage of patients.

Patient Age	Patient Number (n)	Patient Percentage (%)
30-39 years	03 patients	09.68%
40-49 years	10 patients	32.26%
50-59 years	05 patients	16.13%
60-69 years	07 patients	22.58%
70-79 years	06 patients	19.35%

83.33% of the breast reconstruction procedures (n=40 procedures) were done at James Cook University Hospital and 16.67% of the breast reconstruction procedures (n=8 procedures) were done at Friarage Hospital. Types of breast procedures/breast reconstruction procedures presented in our study are detailed in Table 2.

**Table 2:** Breast reconstruction procedures. n: Number of procedures; %: percentage of procedures; LN: lymph node.

Types of Operations	Number of Procedures (n)	Percentages of Procedure (%)
Skin and areola-nipple complex sparing mastectomy plus/minus sentinel LN biopsy	06	12.50%
Skin-sparing mastectomy -excision of areola-nipple complex plus/minus sentinel LN biopsy	20	41.67%
Second stage reconstruction and exchange of implant	20	41.67%
Delayed reconstruction	01	02.08%
Augmentation mammoplasty	01	02.08%

Indications of the breast procedures/breast reconstruction procedures in our study are detailed in Table 3.

**Table 3:** Indications of procedures. n: Number of procedures; %: percentage of procedures.

Indication of Surgery	Number of Procedures (n)	Percentage of Procedures (%)
Risk-reducing mastectomy	09	18.75%
Invasive ductal/lobular breast carcinoma	17	35.42%
Exchange of implant or expander (due to postoperative complications or size change due to patient wish	07	14.58%
Second-stage breast reconstruction	13	27.08%
Cosmesis (delayed reconstruction or augmentation mammoplasty)	02	04.17%

In our study group, the areola-nipple complex was unilaterally sacrificed in 25.0% (n=12), bilaterally sacrificed in 14.58% (n=7), unilaterally preserved in 22.92% (n=11), and bilaterally preserved in 37.50% (n=18). All patients had immediate reconstruction; in 31.25% of the procedures (n=15), an expander was placed, in 43.75% (n=21) of the procedures, an expander was changed to implants, and in 25.0% (n=12) of the procedures, direct implants were placed.

97.92% (n=47) of the implants were placed pre-pectoral, and 02.08% (n=1) of implants were placed sub-pectoral. 20.83% (n=10) of the patients had bilateral procedures.

Out of 31 patients (48 procedures), we achieved a zero percent infection rate within the first 30 days following the procedures. In 12.50% (n=6) of the procedures, patients developed complications post-breast reconstruction.

One patient 02.08% (n=1) who had a single procedure experienced a delayed infection at the implant 47 days after the procedure due to topping up of the expander in an outpatient clinic. A swab from the surgical wound revealed S. aureus with no significant risk factors, which ultimately required the removal of the implant (the patient opted not to have salvage of the reconstruction). This is compared to the rate of breast reconstruction infection from the National Mastectomy and Breast Reconstruction Audit 2011 (25%) is significantly low.

One patient 02.08% (n=1) who had a single procedure developed a mild infection at the drain exit site 11 days post breast reconstruction procedure, which responded completely to an oral antibiotic course and regular dressing. The reconstruction itself was not infected, and the length of the subcutaneous tunnel of the drain was 6-7 cm. A swab from the drain site isolated S. aureus. However, the swab from the surgical wound of the same patient showed no microbial growth. Unfortunately, the drain site was not sutured at the time of the drain removal as per the current protocol due to logistical difficulties.

Two patients 04.17% (n=2) who had one procedure each developed partial necrosis of the skin of the reconstructed breast. One patient 3.23% (n=1) with a prior history of breast radiotherapy required a return to theatre for excision of a necrotic skin patch (1 x 6 cm) and an exchange of the implant. The other patient's necrosis 3.23% (n=1) was managed conservatively without further surgical intervention.

One patient 02.08% (n=1) who had one procedure developed delayed dehiscence of the reconstruction wound on the right breast, necessitating secondary stitching and replacement of the right implant. No microbial organisms were isolated from the wound, and the patient had an uneventful post-operative recovery.

One patient 02.08% (n=1) who had one procedure experienced recurrent large seromas, requiring an exchange of the expander with a new one. Risk factors for this individual included smoking and radiotherapy following wide local excision six years earlier.

Post-breast reconstruction complications are shown in Table 4.

**Table 4:** Post-breast reconstruction complications n: Number of procedures; %: percentage of procedures.

Complications	Number of Procedures (n)	Percentage of Procedures (%)
Delayed infection at the reconstruction site	01	02.08%
Drain exit site infection	01	02.08%
Necrosis of the skin of the reconstructed breast	02	04.17%
Delayed dehiscence of the reconstruction wound	01	02.08%
Large seromas (exchange of the expander)	01	02.08%

## Discussion

SSIs are classified into superficial incisional SSIs, deep incisional SSIs, and organ/space SSIs. Superficial incisional SSIs occur in the first 30 days post-operatively and affect only skin/subcutaneous layers, and their clinical diagnosis is pain, redness, and purulent discharge. Deep incisional SSIs occur within 30 days and can be up to one year post procedure, and fascial planes are generally affected. They are diagnosed by pain, redness, purulent discharge, and incision reopening with infection. Organ/space SSIs occur within 30 days and can be up to one year post procedure, infection is typically beyond fascial planes, with or without implant involvement, and they are diagnosed clinically by purulent discharge, abscess, culture-positive organisms, or physician assessment findings [10].

Staphylococcus species are commonly isolated micro-organisms in SSI patients as recorded by Palubicka et al. [11,12]. In our study, we had one patient who had one procedure (02.08%) and developed a mild infection at the drain exit site. The swab from this site isolated Staphylococcus. aureus. Another patient who had a single procedure (02.08%) presented with a delayed infection at the reconstruction site. Following breast reconstruction surgery using implants, the most isolated organisms post-SSIs are gram-positive strains such as Staphylococcus epidermidis and Staphylococcus aureus, while gram-negative organisms such as Pseudomonas aeruginosa are isolated in only 16% as reported by Piper et al. [10]. Biswal et al. reported that hospital tanks are a major source of infections, like Gram-negative bacteria, particularly Pseudomonas. Consequently, in our study, we ensured that all staff underwent scrubbing with a brush and kept the drape trolley away from the theatre tap to prevent water splashes on sterile drapes, with an aim to minimize infection rates [6].

McCarthy et al. reported that the risk of SSIs and subsequent complications post expander/implant reconstructions are higher among smokers, women over 65, obese patients, and those with hypertension [13]. In our study, one patient (02.08%) who had a single procedure and a history of smoking experienced recurrent large seromas, necessitating expander replacement. Weichman et al. also reported that factors like age over 50, diabetes, hypertension, and obesity are associated with higher infection rates [14]. Reish et al. concluded in a single-centre study of 1952 breast reconstruction procedures using implants that smoking is a major risk factor for implant infection and subsequent reconstruction failure [15].

Timing of reconstruction is crucial, and Alderman et al. reported a higher infection rate of 35.4% as well as higher complication rates post-immediate breast reconstruction procedures. In our study, all patients had immediate reconstruction, in 31.25% of the procedures (n=15), an expander was placed, in 43.75% (n=21) of the procedures, an expander was changed to implants, and in 25.0% (n=12) of the procedures, direct implants were placed. 12.50% (n=6 procedures) of patients developed complications post-breast reconstruction [16].

History of radiotherapy prior to implant reconstruction is associated with higher infection rates of approximately five times as reported by Nahabedian et al. In our study, one patient who had one procedure (02.08%) experienced recurrent, large seromas, leading to the exchange of the expander with a new one. This patient had radiotherapy following wide local excision six years prior to his breast reconstruction procedure. In addition, one patient who had one procedure (02.08%) experienced partial skin necrosis due to a prior history of breast radiotherapy. This patient required a return to theatre for excision of a necrotic skin patch (1 x 6 cm) and an exchange of implant [17]. Similar findings are confirmed by Sbitany et al. and they concluded that any history of radiation increases the infection rates by up to 21.6% [18].

The adjuvant-chemotherapy is associated with a higher infection rate, post-reconstruction procedures in comparison to the neoadjuvant-chemotherapy and no chemotherapy [15].

Perioperative antibiotic prophylaxis prior to immediate breast reconstruction is associated with an infection rate of only 5.8% in comparison to a 14.4% infection rate without perioperative antibiotics prophylaxis [19]. Clayton et al. concluded in their retrospective study that pre-operative antibiotics and post-operative antibiotics up to drain removal in breast reconstruction procedures are associated with a lower infection rate of 18.1% (n=116 patients) from 34.3% (n=134 patients) in those patients who received a single antibiotics dose [20]. Antibiotics are typically administered within 30-60 minutes prior to skin incision and the recommended antibiotics are cephalosporin either first or second-generation or clindamycin in allergic patients [20]. All patients in our study had intravenous antibiotic prophylaxis at the time of anaesthetic induction (Teicoplanin 1.2 g), followed by 400 mg every 12 hours IV for two days.

The risk of skin-flap necrosis is higher on performing one-stage breast reconstruction than two-stage tissue expander procedures, and there is no significant difference in the infection rates between both procedures [21].

All patients in our study had a morning shower with chlorhexidine. If the patient is a known MRSA carrier, it is recommended to use decontamination agents like topical mupirocin and five days of body scrub with chlorhexidine [22].

Skin cleaning prior to incision with alcohol-based solutions (95-110%), chlorhexidine, or iodine can kill the skin commensals, while the combination of alcohol and chlorhexidine is associated with low SSIs by up to 6.4% [23]. In our study, we used a solution of chlorhexidine (2%) (2% chlorhexidine gluconate 70 isopropyl alcohol skin disinfectant) for skin preparation as per the surgeon’s new policy and NICE guidelines. 

Nipple shields are used to reduce bacterial contamination such as S. epidermidis. This has been supported by the findings of the study by Collis et al. which did not show any bacterial presence from above the nipple shield swabs, and only 33% of the swabs showed bacterial presence below the nipple shields, whereas the positive swabs were almost the double in the patient group where nipple shield was not used [24]. We used nipple glue in all patients in our study.

Minimal trauma to skin and breast tissues, meticulous haemostasis, and careful pocket preparation post-mastectomy along with usage of fresh instruments, irrigation with normal saline and then antibiotic or povidone-iodine solution, and soaking the implant in antibiotics solution (50,000 U bacitracin, 80 mg gentamicin, 1 g cefazolin in 500 mL normal saline) have been associated with a reduced infection rate and capsular contracture. In our study, we strictly followed all the above measures, and we prepared an antibiotic solution with 1.2 gm teicoplanin, 240 mg gentamicin, and 1000 mL saline for pocket irrigation [25,26]. This resulted in only one delayed infection from our procedures.

Measures such as minimal handling of the implant or expander, immediate insertion of the implant to decrease environmental exposure, changing the gloves on handling the implant, and minimal skin contact by using retractors or an introduction sleeve are associated with reduced infection rate and have been strictly followed up in our study [25]. We performed closure in layers using monofilaments sutures, and it has been advised to avoid using polyfilament sutures because they harbour bacteria and increase the infection rates [27].

Post-operative drain reduces the infection by draining the excess fluid and, at the same time can facilitate bacterial entrance. Therefore, McCarthy et al. concluded that there is no significant difference in the infection rate if the drain has or has not been used [27]. Measures such as drain tunnelling, reinforcement with Allo-Derm, mupirocin application, and early drain removal are associated with low infection rates and all of the previous measures have been followed up in our study group [28].

The limitation of the study is that it is a retrospective study which makes it subjective to selection bias, and there are other limitations in terms of accuracy, completeness, and consistency. Also, it is a single-centre study and a single-consultant study which makes it again subjected to selection bias than multi-centre studies as this might limit the generalisability of the findings. The study population is relatively small (31 patients and 48 procedures) and the duration of the study is also relatively short (17 months).

## Conclusions

SSIs are still a significant morbidity post-breast surgery, especially in breast reconstruction procedures. Preventing SSIs in implant-based breast reconstructive procedures is always the desirable outcome. At our hospital, we developed the PBRS which has been associated with low infection rates post-operatively (02.08%) in comparison to the current guidelines and published literature (up to 25%). Therefore, we strongly recommend adopting strict adherence to the PBRS to improve post-procedure outcomes. Also, we recommend further research with strict adherence to the PBRS and larger sample sizes to confirm these observations.
